# Accumulation and variability of maize pollen deposition on leaves of European Lepidoptera host plants and relation to release rates and deposition determined by standardised technical sampling

**DOI:** 10.1186/s12302-016-0082-9

**Published:** 2016-04-26

**Authors:** Frieder Hofmann, Maren Kruse-Plass, Ulrike Kuhn, Mathias Otto, Ulrich Schlechtriemen, Boris Schröder, Rudolf Vögel, Werner Wosniok

**Affiliations:** 1 TIEM Integrated Environmental Monitoring, Dortmund/Bremen, Germany; 2 Ökologiebüro, Bremen, Germany; 3 Wölsauerhammer, Marktredwitz, Germany; 4 Büro Kuhn, Bremen, Germany; 5 Federal Agency for Nature Conservation (BfN), Bonn, Germany; 6 Sachverständigenbüro, Dortmund, Germany; 7 Environmental Systems Analysis, Institute of Geoecology, Technische Universität, Brunswick, Germany; 8 Berlin-Brandenburg Institute of Advanced Biodiversity Research (BBIB), Berlin, Germany; 9 Agency for Environment, Health and Consumer Protection, Eberswalde, Brandenburg Germany; 10 Institute of Statistics, University of Bremen, Bremen, Germany

**Keywords:** *Zea mays*, *Bt* maize, Exposure, Genetically modified organisms (GMOs), Risk assessment, Risk management, Pollen, Leaf deposition, Butterfly, Lepidoptera, Host plants

## Abstract

**Background:**

Risk assessment for GMOs such as *Bt* maize requires detailed data concerning pollen deposition onto non-target host-plant leaves. A field study of pollen on lepidopteran host-plant leaves was therefore undertaken in 2009–2012 in Germany. During the maize flowering period, we used in situ microscopy at a spatial resolution adequate to monitor the feeding behaviour of butterfly larvae. The plant-specific pollen deposition data were supplemented with standardised measurements of pollen release rates and deposition obtained by volumetric pollen monitors and passive samplers.

**Results:**

In 2010, we made 5377 measurements of maize pollen deposited onto leaves of maize, nettle, goosefoot, sorrel and blackberry. Overall mean leaf deposition during the flowering period ranged from 54 to 478 n/cm^2^ (grains/cm^2^) depending on plant species and site, while daily mean leaf deposition values were as high as 2710 n/cm^2^. Maximum single leaf-deposition values reached up to 103,000 n/cm^2^, with a 95 % confidence-limit upper boundary of 11,716 n/cm^2^.

**Conclusions:**

Daily means and variation of single values uncovered by our detailed measurements are considerably higher than previously assumed. The recorded levels are more than a single degree of magnitude larger than actual EU expert risk assessment assumptions. Because variation and total aggregation of deposited pollen on leaves have been previously underestimated, lepidopteran larvae have actually been subjected to higher and more variable exposure. Higher risks to these organisms must consequently be assumed. Our results imply that risk assessments related to the effects of *Bt* maize exposure under both realistic cultivation conditions and worst-case scenarios must be revised. Under common cultivation conditions, isolation buffer distances in the kilometre range are recommended rather than the 20–30 m distance defined by the EFSA.

**Electronic supplementary material:**

The online version of this article (doi:10.1186/s12302-016-0082-9) contains supplementary material, which is available to authorized users.

## Background

Maize is wind-pollinated and releases prodigious quantities of pollen into the environment during anthesis [1]. In the case of genetically modified *Bacillus thuringiensis* (*Bt*) maize, this pollen also carries insecticidal *Bt* protein. Because non-target herbivores may feed on host plants dusted with *Bt* pollen—both within and around maize fields, this exposure and its subsequent effects must be evaluated to assess the risks associated with the cultivation of *Bt* maize.

Risks posed to non-target Lepidoptera by the cultivation of *Bt* maize plants resistant to lepidopteran species such as the European corn borer are widely acknowledged [2–5]. Scientific controversies exist, however, in regard to estimates of the potential magnitude of effects and measures such as isolation buffer distances applied for risk management [5–14].

Because exposure is the basis of any effect, the interpretation of maize pollen leaf deposition data is highly relevant to the above debate. For several reasons, successful interpretation is non-trivial. To date, virtually all measurements of leaf pollen deposition—both direct measurements of pollen deposition on leaves and indirect measurements (other acceptors, technical instruments, proxy parameters)—have been obtained by non-standardised methods that differ in methodology, time span and available background information on the quantity of pollen shedding. It is therefore difficult, if not impossible, to compare these data directly [15]. In addition, some commonly used methods, such as the use of adhesive slides in open exposure, are non-standardised methods regarded as semi-quantitative [16–18]. Although standardised methods such as Durham trap [16] and pollen mass filter (PMF) [19] approaches were described as early as the 1940s, only a few authors have used such techniques to facilitate data comparison. Similarly, few researchers have determined pollen release rates over the flowering period using standardised volumetric pollen monitors such as the Hirst trap [20] or the PMO [10].

Most studies and available data on leaf deposition have been related to mean values of leaf surface pollen density over varied time periods. For instance, the results of Stanley-Horn et al. [21], Pleasants et al. [22] and Dively et al. [23], obtained by various methods, were mainly collected daily or cumulatively over sampling periods ranging from 3 to 14 days. Other authors, not recognising that pollen deposition is a cumulative process, have concentrated solely on daily deposition rates as a measure of risk [24–26].

Pollen deposition onto surfaces such as plant leaves is the result of a complicated input–output process [27]. Although slides used for measurement of daily deposition rates are usually replaced daily, leaves remain on plants and deposition continues as long as pollen is emitted. Accumulation on the leaf surface thus takes place over time, with lepidopteran larvae correspondingly exposed to an integrated deposition on the leaf surface rather than to single daily deposition rates. Relocation and spatial aggregation processes take place on the leaf surface as well. Losses occur through rain and wind movement, thereby causing the distribution of settled pollen on a leaf to become increasingly inhomogeneous. Because deposition is a cumulative process, precision regarding the nature of collected data is crucial. Leaf deposition calculated as pollen density on a leaf at time *t*, which reflects the balanced result of input–output processes, must be distinguished from daily deposition rates, both in regard to daily input as well as cumulative rates of deposition over time. Although described in the aerobiological literature [17, 18, 27–31], this process and its context have not been adequately taken into account in some publications addressing genetically modified organism (GMO) risk assessment [7, 8, 11, 12, 14].

The first comprehensive study of maize pollen emission, transport and deposition was reported by Raynor et al. [28, 29] in the 1970s, decades before the advent of GMOs. Using small-scale experimental fields (0.26 ha), they observed average daily deposition rates as high as 250 pollen grains per square centimetre per day [n/(cm^2^ day)] near the source, with maximum daily rates up to 657 n/(cm^2^ day) over 14 days during anthesis. In regard to total deposition over the flowering period, they detected an average value of 1260 n/cm^2^ close to the field source. When the authors attempted to record the maximum possible deposition for worst-case assessments, they found it difficult to maintain a permanent downwind position during field measurements because of changing wind directions. They therefore provided recommendations for correction of this observational error using detailed measurements of pollen release rates, meteorological parameters and dispersal modelling.

Kawashima et al. [30] followed this directive during simultaneous measurements of daily pollen deposition rates and meteorological data over a 19-day flowering period. To measure airborne pollen and pollen release rates, they used a standardised Durham sampler. This device measures sedimentation in terms of dry deposition, which can be related on the basis of Stokes’ law to air pollen concentration and corresponding release rates. To determine plant-specific deposition onto exposed leaves, the authors used open sticky slides. They detected airborne pollen daily sedimentation rates and daily deposition rates on exposed leaves (daily input rates) of up to 1710 n/(cm^2^ day) (Figs. 2 and 4, respectively, in [30]), with corresponding integrated (cumulative) leaf deposition over the flowering period of approximately 7400 n/cm^2^. Because of changes in wind direction, a leeward position could not be maintained on all days. Following the recommendations of Raynor et al. [28, 29], they obtained an average value of 2.0 for the overall ratio of measured to potential deposition assuming a permanent leeward position. Taking into account the worst-case risk assessment scenario, typically done by assuming a permanent leeward position, they then calculated the integrated potential deposition over the flowering period to be approximately 15,000 n/cm^2^.

A concise risk assessment model for *Bt* maize and European Lepidoptera published by Perry et al. [7, 8, 11, 12] has been incrementally adopted in several stages for EU risk assessment [2–4, 14, 32–34]. Because of a lack of adequate empirical data, however, the exposure portion of the model is based partly on expert opinion instead of actual data [8, 14]. To estimate exposure, the original model was based on a small dataset provided by Wraight et al. [35], who placed Vaseline-coated slides adjacent to a maize field for 7 days and recorded a pollen density of up to 221 n/cm^2^. Assuming these data for leaf deposition and worst-case exposure of lepidopteran larvae (*Inachis io* on *Urticadioica* leaves), a mean pollen density at the field edge of 66.3 n/cm^2^ over 7 days and on leaves of 9.5 n/cm^2^ per day was calculated by Perry et al. [8, Appendix]. Because pollen grains are not uniformly distributed on a leaf, some segments of the larval population will encounter pollen densities higher than average with a certain likelihood. To correctly assess their exposure, data on pollen distribution on leaves are needed, an aspect generally accepted in the risk assessment [7, 22]. Expressing pollen deposition in units of n/cm^2^, Perry et al. [7] used a leaf area size of 0.03 cm^2^ as a reference, as the feeding behaviour of young larvae is important. This leaf area size, which was based on laboratory-conducted toxicity studies of Felke et al. [36], ensured that a leaf with applied pollen was consumable in 1 day even by young larvae. To account for inhomogeneous deposition of pollen on leaves, including that of aggregation processes, Perry et al. [7] assumed a factor of 12 for a 99 % confidence interval (CI) around mean pollen densities; for worst-case field scenario assumptions, they estimated a mean pollen density of approximately 599 n/cm^2^ on nettle leaves and a daily mean density of 85 n/(cm^2^ day) [8, Appendix I]. The European Food Safety Authority further developed the original model in several stages: expert judgements on 11 sources of uncertainty were included and various exposure assessment scenarios were estimated, with resulting risks all ranging below those of the original model in each case [14].

Field data on the variability of pollen deposition onto leaves, especially data with adequate spatial resolution, are currently lacking [14]. To realistically assess leaf deposition variability, such information is necessary. This requirement is particularly true for worst-case considerations, in which potential maximum exposure levels are also relevant.

The primary aim of this study was to close this information gap by analysing the variability of maize pollen deposition onto leaves through in situ measurements with detailed spatial and temporal resolution. To achieve this goal, we applied a newly developed in situ pollen microscopic method [15] during a comprehensive field study [37].

Another important focus of this study was the combination of plant-specific leaf pollen deposition data with pollen release and deposition rates measured by standardised technical pollen sampling methods. Relationships can only be properly analysed by simultaneously measuring these parameters. To perform a detailed assessment of pollen release and deposition rates, we used a volumetric pollen monitor (PMO) to continuously measure aerial pollen concentrations in maize fields at the height of the pollen-emitting tassels. We also measured the integrated deposition of pollen inside and outside of maize fields with standardised passive samplers (PMFs). Using this approach, we generated a unique, previously unavailable dataset to estimate the exposure of non-target organisms to *Bt* maize pollen.

## Results and discussion

In this study, we made 5377 measurements of maize pollen on leaf surfaces of maize and other butterfly host plants growing within and adjacent to two maize fields during the main maize flowering period from mid-July to the second half of August in 2010. The recorded data consisted of 2497 measurements on maize, 1646 on nettle (*Urtica dioica*), 586 on goosefoot (*Chenopodium album*), 324 on sorrel (*Rumex acetosa*) and 324 on blackberry (*Rubus* sp.). These measurements and additional ones obtained between 2009 and 2012 were taken near Angermünde, Brandenburg, Germany. To our knowledge, this is the first in situ study showing the variability of leaf deposition data to this extent and level of detail.

A collection of photographs taken of maize leaves during the flowering period illustrates the variability of pollen deposition onto leaves. As seen in Additional file 1: Figure S1 for an example, maize pollen, visible as a yellow powder on leaves, exhibited various deposition patterns, thus showing an inhomogeneous distribution. Pollen grains tended to aggregate and adhere to leaf structures such as midribs, veins and leaf bases, consistent with observations by other authors [26]. This aggregation was not restricted to such structures, however, as clumps of pollen were present all over the leaf surface.

A detailed microscopic-scale inspection confirmed the observed variability of pollen deposition onto leaves (see Additional file 1: Figure S2 for an example).

### Leaf deposition data

**Fig. 1:**
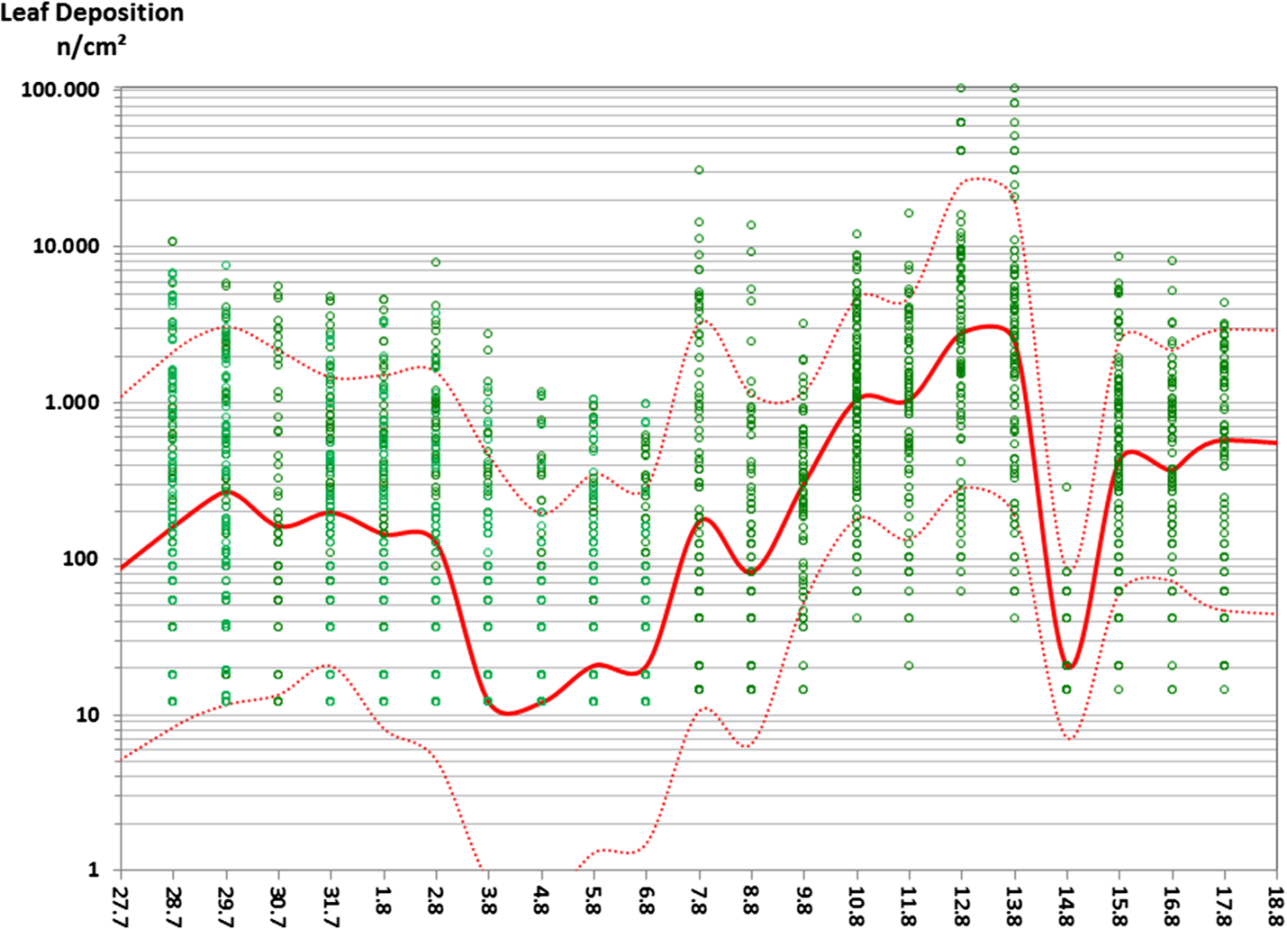
Maize (*Zea mays*) pollen deposition onto maize leaves during the 2010 flowering season. Shedding of pollen from an early flowering maize variety began in field A on 23 July and from a later-flowering variety in field B on 3–4 August. The maize flowering period ended around 23 August. Leaf deposition measurements in 2010 were made in field A from 28 July to 6 August and in field B from 7 to 17 August. *Green dots*, data (*n* = 2497); *solid red line*, daily mean; *red dashed lines*, 90 and 10 % quantiles based on the daily data and the censored maximum likelihood-fitted log-normal distribution

Individual pollen deposition measurements over time, together with daily means and 90 and 10 % quantiles, are plotted in Fig. 1 for maize leaves, Additional file 1: Figure S3 for nettle, and Additional file 1: Figure S4 for goosefoot, sorrel and blackberry. To account for values below detection limits, censored maximum likelihood (CML) was used to estimate the means and quantiles shown in Fig. 1, Additional file 1: Figure S3, Additional file 1: Figure S4. (Means in the figures are geometric means, equivalent to the CML 50 % quantile and median). Flowering began in early maize field A on 23 July 2010, with leaf measurements first taken on 29 July. Pollen deposition was highly variable around daily means and from day to day until the end of the measurement period on 17 August. Deposition values exceeding 1000 n/cm^2^ were regularly measured, with numbers above 10,000 n/cm^2^ frequently recorded. Observed daily mean pollen densities on leaves were as high as 2710 n/cm^2^ for maize and 1665 n/cm^2^ for nettle. Maximum single leaf-deposition values were 103,000 n/cm^2^ on maize and 13,802 n/cm^2^ on nettle (field B).

Whereas observed daily mean values overlapped with the upper range of literature values, maximum single leaf-deposition values greatly exceeded those previously reported. The reason for this discrepancy is that mean leaf values in most other studies were measured on leaf surfaces without further spatial resolution and/or only daily deposition rates were recorded (e.g. on slides)—and only on one or a few days of the flowering period. Some authors even excluded higher deposition values at aggregation zones, such as around midribs [11, 22]. In contrast, our measurements revealed all the variation in leaf deposition values in situ over the main flowering period.

Previously reported maximum pollen deposition values are up to 1710 n/(cm^2^ day) for daily deposition rates, 7400 n/cm^2^ for measured cumulative deposition on slides [30] and higher than 1000 n/cm^2^ for mean leaf deposition (e.g. 1449 n/cm^2^ [22] and 3000 n/cm^2^ [15]).

Dively et al. [23] detected mean deposition values up to 651 n/cm^2^ on *Asclepias* spp. leaves. Hansen Jesse et al. [38] measured values on *Asclepias syriaca* leaves as high as 506 n/cm^2^ within maize fields and 427 n/cm^2^ at 0.2 m from the edge. On the same plant species, Stanley-Horn et al. [21] reported levels up to 429 n/cm^2^ after 11-day exposure in a maize field in Ontario. Zangerl et al. [26] recorded a maximum of 320 n/cm^2^ on leaves of *Pastinaca sativa* located 0.5 m from the field edge. Schuppener et al. [25] detected up to 212 n/(cm^2^ day) on *Urtica* leaves close to the edge of the field; however, that study has been criticised as methodologically unrepresentative [39] and biased towards excessively low values. Lang et al. [24, 40] detected daily deposition rates of up to 429 n/(cm^2^ day) on slides, with significantly lower levels (31 ± 14 %) found on leaves of *Daucus carota.* Gathmann et al. [41] reported higher levels: up to 972 n/cm^2^ on *Chenopodium album* and 894 n/cm^2^ on *Sinapis alba* at a distance of 1 m from the field edge. Kawashima et al. [30] measured daily deposition rates as high as 1710 n/(cm^2^ day) on slides; Shirai and Takahashi [42] recorded levels up to 624 n/(cm^2^ day). During a 10-day rainless period in Iowa, Pleasants et al. [22] found that the highest values in the field ranged from 752 to 1.449 n/cm^2^ on *A. syriaca* leaves. In that study, however, high deposition values at aggregation zones around midribs were excluded from the risk assessment on the basis of a single personal communication [22] that young butterfly larvae do not eat midribs [22 (p. 11, 921), 43]. Lang and Otto [44] have recently reported that such a general assumption is not justified: larvae of the small tortoiseshell butterfly (*Aglais urticae*) have been observed to consume leaf-ribs, including midribs, although they feed on the latter to a lesser extent.

### Statistical description of leaf deposition data

Based on CML estimates from the fitted log-normal distribution, the (geometric) mean of pollen deposition onto leaves over all sites and days ranged—depending on plant species—between 54 and 478 n/cm^2^; standard deviations varied from 0.58 to 1.03 on a logarithmic scale, which is equivalent to a factor of 3.8 to 10.7 on a linear scale (Table 1). Observed maxima of single values reached 2246–103,000 n/cm^2^, depending on plant species and site. For risk assessment (e.g. for consideration of worst-case estimates) the 95 and 90 % quantiles are of great interest, as they reflect levels exceeded in only 5 and 10 %, respectively, of all cases. The 95 % quantile ranged between 953 and 11,716 n/cm^2^, the 90 % quantile between 547 and 5780 n/cm^2^.

The maximum daily mean pollen density recorded on leaf surfaces in 2010 was 2710 n/cm^2^ on maize leaves. On 2 out of 11 days, the daily mean deposition exceeded 1000 n/cm^2^; on another 4 days, it was above 500 n/cm^2^. During preliminary measurements in 2009, we observed mean leaf deposition values on maize as high as 3000 n/cm^2^ on several days [15].

**Table 1:** Statistical parameters of leaf deposition data

Maize pollen deposition on leaves in n/cm^2^		Maize *Z. maize*			Nettle *U. diocia*			Goosefoot *C. alba*	Sorrel *R. acetosa*	Blackberry *Rubus sp.*
Field/site		Total	A	B	Total	A	B	B	A2	A1
Sample size n	5377	2497	1593	904	1646	810	836	586	324	324
Parameters and quantiles from the fitted log-normal-distribution (CML estimate)										
Mean _log10_	µ_log10_	2.12	1.79	2.68	2.14	1.89	2.39	2.57	1.73	2.20
SD._log10_	σ_log10_	1.03	1.01	0.84	0.74	0.66	0.72	0.68	1.01	0.58
SD factor on linear scale	σ_lin Factor_	10.7	10.3	7.0	5.5	4.6	5.3	4.8	10.3	3.8
95 % quantile	L95 %	6497	2831	11,716	2270	953	3791	4908	2499	1452
90 % quantile	L90 %	2747	1214	5780	1225	547	2067	2774	1070	892
75 % quantile	L75 %	652	295	1775	437	217	751	1070	260	395
50 % quantile	L50 %	132	61	478	139	77	244	371	54	160
25 % quantile	L25 %	27	13	129	44	28	79	129	11	65
10 % quantile	L10 %	6.3	3.1	40	16	11	29	50	2.7	29
5 % quantile	L5 %	2.7	1.3	20	8.5	6.3	16	28	1.2	18
Maxima and quantiles from the empirical distribution function										
Maximum	Emax	103,000	14,112	103,000	13,802	10,987	13,802	17,098	6055	2246
95 % quantile	E95 %	4613	2282	8858	2194	879	3770	4532	2043	1213
90 % quantile	E90 %	2526	1152	5090	1152	586	2078	3389	1270	864
75 % quantile	E75 %	762	342	1730	474	244	700	989	391	488
50 % quantile	E50 %	162	72	536	146	49	288	412	49	169
25 % quantile	E25 %	36	18	124	33	<33	82	144	33	49

Results of the application of different statistical models to the collected deposition data. The log-normal distribution was fitted to the data by censored maximum likelihood (CML) estimation. The empirical distribution describes the raw data

The maize pollen leaf-deposition data varied over a certain range. In Additional file 1: Figure S5a–g, this variation is presented as a probability density function for each plant species, with respective cumulative distribution functions displayed in Additional file 1: Figure S6.

To describe the level and random variation of pollen deposition onto leaves, a log-normal distribution was fitted to the observed data using a CML approach. This method was used to account for observational detection limits. The geometric mean of the fitted distribution, which is also the median, was used to characterise the deposition level. Random variation was described by quantiles of the fitted distribution.

*Maize* The distribution of pollen on maize leaves, which was based on the complete dataset from both fields in 2010, is shown in Additional file 1: Figure S5a. Using the CML approach, we estimated an overall mean of 132 n/cm^2^. The 90 and 95 % quantiles were 2747 and 6497 n/cm^2^, respectively. The density of pollen deposition in late-flowering field B is shown in Additional file 1: Figure S5b. The flowering in this field occurred in a concentrated fashion over more than 20 days. The CML estimate for the overall mean was 478 n/cm^2^, which was higher than that calculated from the data for the entire flowering period over both fields. The 90 and 95 % quantiles were 5780 and 11,716 n/cm^2^, respectively.

*Nettle* As shown in Additional file 1: Figure S5c, the mean density of deposited pollen on *Urtica* leaves calculated from all data from both fields was 139 n/cm^2^. The 90 and 95 % quantiles were 1225 and 2270 n/cm^2^, respectively. Similar to maize, a marked increase in maximum daily pollen deposition was evident when data only from the late-flowering field were analysed (Additional file 1: Figure S5d). In this case, mean CML-estimated pollen leaf deposition was 244 n/cm^2^. The quantile values, 2067 n/cm^2^ (90 % CI) and 3791 n/cm^2^ (95 % CI), also considerably exceeded respective values from data covering both fields and the complete sampling period.

*Goosefoot* The maize pollen deposition data for *Chenopodium* leaves (Additional file 1: Figure S5e), acquired from plants growing in field B, exhibited a CML-estimated mean of 371 n/cm^2^ and a standard deviation σ_log10_ of 0.68 (i.e. a factor of 4.80 on a linear scale). The 90 and 95 % quantiles were 2774 and 4908 n/cm^2^, respectively.

*Blackberry* The probability distribution of deposited pollen on *Rubus* leaves (field A, Additional file 1: Figure S5f; CML) displayed a mean of 160 n/cm^2^ with a σ_log10_ of 0.58 (a factor of 3.82 on a linear scale). Quantile values were determined to be 892 n/cm^2^ for the 90 % CI and 1452 n/cm^2^ for the 95 % CI.

*Sorrel* Pollen deposition onto *Rumex acetosa* leaves (field A, Additional file 1: Figure S5g; CML*)* showed a mean of 54 n/cm^2^ with a σ_log10_ of 1.01 (a linear factor of 10.3). Quantile values were 1070 n/cm^2^ at 90 % and 2499 n/cm^2^ at 95 %.

Using the CML estimates, mean leaf deposition ratios between the various species and maize (on a linear scale) as well as ratios of their corresponding standard deviations (in parentheses, for σ on a log_10_ scale) were as follows: *Urtica dioica* 1.05 (0.72) [total], 1.26 (0.65) [field A], 0.51 (0.86) [field B]; *Chenopodium album* 0.78 (0.81) [field B]; *Rumex acetosa* 0.89 (1.0) [field A]; *Rubus* sp. 2.62 (0.57) [field A].

An analysis of the frequency distribution of maize pollen deposition onto *A. syriaca* leaves was previously attempted by Pleasants et al. [22]. Their analysis was based on an extensive study at four sites in the US over several years. Because the authors frequently made three 1-cm^2^ sub-measurements per leaf, variability on the leaf surface could be assessed to some extent. In addition to the fact that three sub-measurements per leaf and a resolution of 1 cm^2^ are insufficient to represent leaf surface variability [15], they selectively excluded midrib zones with high deposition values at most of their study sites (see above). The resulting frequency distribution was consequently not representative of the deposition of pollen onto leaves. On the basis of carefully performed sample measurements, they estimated that the actual deposition was higher by a factor of 1.5–1.9 when midribs were included. The mean leaf deposition values calculated by taking this factor into account lie within the range of our results, although our range of single leaf-deposition values far exceeds that of their data.

If technically acquired deposition data is also treated as a proxy for leaf deposition, our results support the findings of Kawashima et al. [30], who measured a cumulative leaf deposition value of around 7400 n/cm^2^ and estimated an integrated potential deposition of 14,791 n/cm^2^ as the worst case under a permanent downwind assumption. Our observations are in good agreement with the values of Kawashima et al. [30] and contradict the assessment of Perry et al. [8], who judged those values to be unrealistically high.

### Relationship between plant-specific pollen deposition onto leaves versus pollen concentration and deposition measured by standardised technical methods

#### Pollen release and deposition

Apart from the effects of pollen shedding and dispersal and pollen grain behaviour, deposition is also acceptor-dependent. Pollen deposition onto leaves is thus specific to plant species and morphology. Plant growth and environmental conditions influence pollen density as well. These factors collectively cause pollen deposition onto leaves to be highly variable, complicating the interpretation of observed deposition. To obtain pollen exposure data that are comparable, additional standardised measurement methods must be used to record aerial pollen concentration and deposition at a given site [19, 45–48]. In this study, we performed parallel measurements of pollen concentration and deposition using standardised technical sampling methods involving a PMO volumetric pollen monitor [10, 37, 39] and PMF passive pollen samplers [9, 19, 47]. These parallel measurements allowed plant-specific leaf deposition to be linked to pollen release, aerial concentration and deposition. The parallel measurements were conducted in later-flowering field B, where anthesis began on 3 August.

**Fig. 2:**
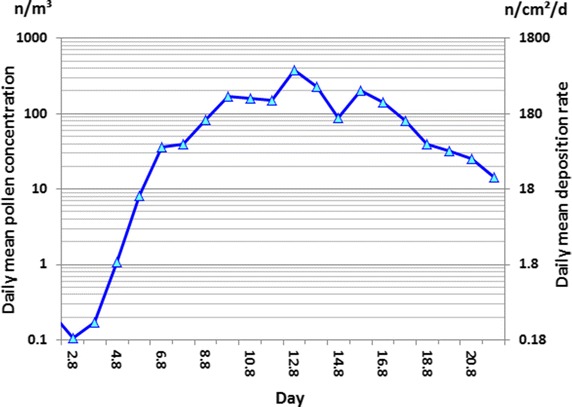
Daily mean pollen concentrations (n/m^3^) indicating pollen release rates and related daily mean pollen deposition rates [n/(cm^2^ day)]. Continuous measurements of pollen concentrations were made by a PMO volumetric pollen monitor at tassel height in late-flowering maize field B near Angermünde in 2010.* Left y-axis*, daily mean pollen concentration at tassel height in the field in n/m^3^ (log_10_);* right y-axis*, daily mean deposition rate in n/(cm^2^ day) (log_10_)

Field measurements of daily mean pollen concentration served as an indicator of daily mean pollen release rates over the main flowering period up to 21 August (Fig. 2), with the corresponding daily deposition rate *D*_d_ derived using Eq. 9. As illustrated in Fig. 2, the rate of pollen release increased rapidly, with the daily mean pollen concentration, *C*_*d*_, in the air rising to a maximum value of 356 n/m^3^ and *D*_*d*_ reaching as high as 615 n/(cm^2^ day).

Similar to previous studies [10, 37], we discerned a distinct diurnal pattern to pollen release, beginning in the early morning with a peak around midday. Furthermore, we observed that pollen release could be interrupted by unfavourable weather conditions. Maximum hourly pollen concentrations in the air reached values higher than 1700 n/m^3^, which is about 4.7 times the level of corresponding daily means.

In field B, flowering and pollen shedding lasted for at least 20 days. This observation disproves a major assumption of the EU exposure assessment, which based its worst-case scenario on a 7-day-long incident, and implies that the flowering period duration of a single field has been underestimated by a factor of approximately 3. A flowering duration of 3–6 weeks has also been demonstrated for maize under commercial cultivation via long-term measurements of aerial pollen concentrations at a reference station in northern Germany over 16 years [10], with various fields differing in their flowering behaviour and influencing deposition at each site in this region. A systematic survey in Bavaria, southern Germany, found a maize flowering period duration of 6–8 weeks on a regional basis [40].

### Comparison of leaf deposition with daily deposition rates, cumulative deposition and standardised integrated deposition

**Fig. 3:**
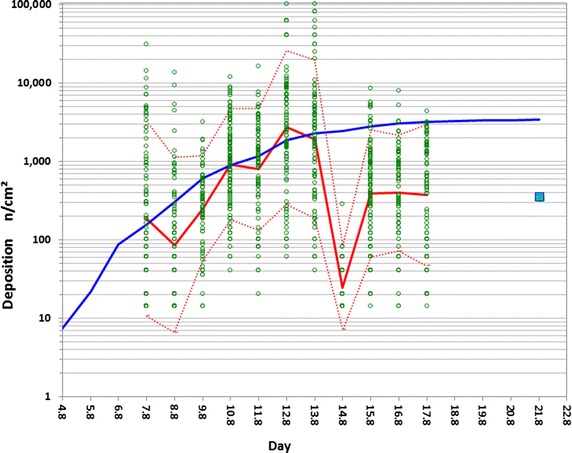
Comparison of mean leaf deposition and its variability with PMF-based cumulative deposition and integrated deposition over the flowering period. Data are from late-flowering field B in 2010. *Green dots*, single leaf-deposition observations; *red line*, daily mean leaf deposition; *dotted red lines*, leaf-deposition 90 and 10 % confidence intervals based on censored maximum likelihood estimation; *blue line*, cumulative deposition rates based on PMO pollen monitor standardised measurements; *cyan square*, integrated deposition based on PMF passive sampler standardised measurements

A log-scale comparison of pollen deposition on maize leaves versus cumulative deposition rates and standardised integrated deposition is shown in Fig. 3. Additionally, Additional file 1: Figure S7 shows this relationship on a linear scale for daily mean leaf pollen deposition values.

We first examined trends in mean leaf-deposition values. Shortly after the beginning of anthesis, daily mean leaf deposition mirrored cumulative deposition, reaching its maximum level of 2710 n/cm^2^ on 12 August and greatly exceeding the maximum daily deposition rate of 678 n/(cm^2^ day). Summer rainfall was frequent during this period. After a strong thunderstorm with heavy precipitation (>40 l/m^2^) on 13–14 August 2010, deposition onto leaves dropped rapidly but then immediately started increasing again. The standardised integrated pollen deposition over the flowering period, *D*_S_ (denoted as a cyan square in Fig. 3), was measured by the PMF technical sampling method that had been standardised according to VDI-standard 4330-3 [19] and CEN-TS 16,817-1 [47]. Because of a sampling efficiency below 1, the absolute value of *D*_S_ determined by the PMF was lower than the total cumulative deposition calculated as the sum of daily rates. For this standardised method, data are available showing the variability between sites and years based on 216 sites and 10 years [9]. The expected average mean deposition for in-field conditions has been found to be 326 n/cm^2^, with a 95 % CI of the mean regression line between 246 and 431 n/cm^2^. The corresponding 95 % CI for single values ranges from 44 to 2388 n/cm^2^. In our study, standardised deposition measurements using the PMF at the site of the leaf measurements gave a value of 359 n/cm^2^. This result indicates that the intensity of pollen deposition at our study field site, while slightly higher than the expected mean value, was well within the average range of variability observed in maize under commercial cultivation in Germany.

In addition to daily mean leaf deposition, the variability of the leaf deposition data and respective 90 and 10 % quantiles illustrated in Fig. 3 demonstrates the wide variation in pollen densities found on leaf surfaces. As shown in the figure, observed daily mean leaf deposition mirrored cumulative deposition rates. Single density values on leaf surfaces showed high variation around the mean leaf deposition.

The empirical data confirm what aerobiology postulates: individual daily deposition rates are insufficient as a measure of daily exposure. Summation of daily deposition rates to give an integrated deposition over time, in contrast, reveals the strong relationship of this calculated value to the development of the daily mean leaf deposition data. A high variation in pollen densities is found on leaf surfaces. Single values can greatly exceed the mean input from the air, demonstrating the importance of variation and relocation processes on and between leaves. Our results also demonstrate that an integrated potential deposition of around 15,000 n/cm^2^ estimated by Kawashima et al. [30] is not unrealistic for a worst-case assessment of exposure. This value lies well within the variation observed for single pollen deposition data on leaf surfaces in our field experiments.

### Combining leaf deposition data with standardised technical measurements for comparable assessment

To obtain comparable data, specific leaf deposition data must be complemented with parallel pollen measurements acquired using standardised technical sampling methods. By combining plant species- and site-specific leaf deposition measurements with data gained by standardised technical measurements, we then can generalise expected exposure over sites and years.

**Fig. 4:**
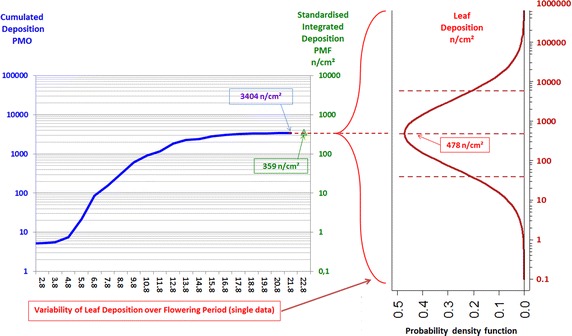
Combining plant-specific leaf deposition and its variability with standardised measurements of deposition. *Blue* cumulative maize pollen deposition, *D*_total_ (=cumulative daily deposition rate), measured with a PMO pollen monitor; *green* standardised integrated deposition, *D*_S_, measured with a PMF passive sampler; *red* variability of plant-specific leaf deposition on maize leaves over the flowering period (log-normal distribution; *red curve*) with mean and 90 % confidence intervals

This procedure is outlined in Fig. 4 for our data. The variability of pollen deposition onto maize leaves is displayed on the right-hand side of the figure (red curve and scale). Although leaf deposition naturally cannot be predicted on a day-to-day basis, the estimated log-normal distribution for deposition provides the probabilities of leaf deposition values over the flowering period. This variability in leaf deposition at the measurement site is related to the standardised measurements of pollen deposition displayed on the left-hand side of the figure. The pollen maize-leaf deposition data are associated via their CML-based mean to both parameters of standardised deposition measurements: total deposition (*D*_total_) and standardised integrated deposition (*D*_S_). The first relationship, that of mean maize-leaf deposition and *D*_total_ (the integrated deposition obtained from measurements of aerial pollen concentration using the PMO pollen monitor; blue curve and scale), is expressed by Eq. 13, leading to a conversion factor of 0.14. As pollen accumulates on a surface during deposition (i.e. over the flowering period), the correct parameter to indicate the intensity of pollen deposition at a site is the cumulative or integrated total deposition over the flowering period (*D*_total_), not any daily rate mean. The second relationship, which links leaf deposition to *D*_S_ measured by the PMF passive sampler, is described by Eq. 14, with a resulting conversion factor of 1.33 (green triangle and scale). By adjusting the scales of the vertical axis of the various parameters in the figure using these factors, the variability of leaf deposition indicated by the CML-estimated distribution can be linked to the pollen deposition values as measured by standardised technical methods, thereby allowing for comparable results.

Corresponding relation factors for maize and nettle mean leaf deposition, along with 90 and 95 % quantiles as indicators of the variation, are given in Table 2. Values higher than the upper 95 % quantile are relevant for worst-case assessments.

**Table 2:** Conversion factors between plant-specific leaf deposition and standardised measurements of pollen deposition

Conversion factors		Standardised technical methods	
Specific leaf deposition		Integrated deposition PMO {3404 n/cm^2^}	Standardised deposition PMF {359 n/cm^2^}
Maize (*Zea mays*)			
Mean (50 % quantile)	$\bar{D}_{\text{ZM}}$ (50 %) {478 n/cm^2^}	0.14	1.33
90 % quantile	D_ZM_ (90 %) {5780 n/cm^2^}	1.70	16.1
95 % quantile	D_ZM_ (95 %) {11,716 n/cm^2^}	3.44	32.6
Nettle (*Urtica dioica*)			
Mean (50 % quantile)	$\bar{D}_{\text{UD}}$ (50 %) {244 n/cm^2^}	0.07	0.68
90 % quantile	D_UD_ (90 %) {2067 n/cm^2^}	0.61	5.76
95 % quantile	D_UD_ (95 %) {3791 n/cm^2^}	1.11	10.5

Specific leaf deposition was estimated by censored maximum likelihood with data obtained from parallel measurements in field B over the flowering period

### Expected variability of leaf deposition under commercial cultivation

The above-mentioned relationships between standardised and plant-specific leaf deposition are particular to our observations at the measurement site. By combining leaf deposition data with PMF-acquired standardised technical measurements, exposure can be assessed in a more general manner.

**Fig. 5:**
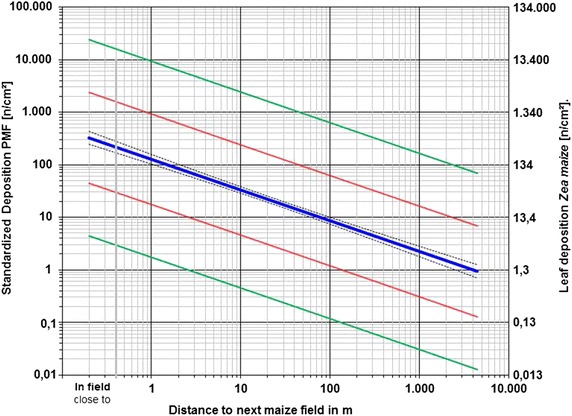
Expected mean and variability of maize pollen deposition onto maize leaves in relation to distance. *Solid* and *dotted blue lines* mean regression line with 95 % confidence interval, respectively; *red lines* 95 % confidence interval for PMF-measured standardised deposition single values; *green lines* 95 % confidence interval for plant-specific (maize) deposition single values

In regard to standardised measurements obtained with the PMF, representative results for maize pollen deposition in relation to distance to the nearest field have been published [9]. The results of the regression of this standardised PMF deposition data are shown in Fig. 5, which includes the expected mean deposition (blue line), 95 % CI of the mean regression (black dotted lines) and 95 % CI for single values (red lines).

As displayed in Fig. 5, expected mean and variability of leaf deposition over sites and years can be estimated by applying our previously calculated PMF-*D*_S_ relation factor to the standardised leaf deposition data (left-hand side) to obtain plant-specific deposition values (right-hand side). In this example, the resulting expected mean value of leaf deposition close to the source (in-field conditions) is estimated to be 434 n/cm^2^ (326 n/cm^2^ × 1.33). The combined variability of within-site leaf deposition data and across-site standardised measurements can be determined using Eq. 15. The 95 % CI calculated for this example, displayed in Fig. 5 as green lines, has single-value lower and upper boundaries of 6 and 31,800 n/cm^2^, respectively, corresponding to a factor of 73 around the mean on a linear scale. As another example, mean leaf deposition close to the source is estimated to be 222 n/cm^2^ (326 n/cm^2^ × 0.68) for nettle. The 95 % CI for nettle ranges between 5 and 10,024 n/cm^2^, a factor of 46 around the mean on a linear scale.

Our measurement data for a realistic assessment indicate, that the mean leaf density expected at 1000 m distance is 2.96 n/cm^2^ (Fig. 5) for maize and 1.52 n/cm^2^ for nettle. This exceeds any exposure level (maximum 0.28 n/cm^2^) related to the defined threshold protection levels (0.5; 1.0 %) assumed in the various EFSA scenarios [14] which were the base for the 20–30 m isolation buffer distances as mitigation measures.

An estimate of the variability of leaf deposition data around the mean based on the variability of PMF measurements [48] has already been provided by Perry et al. [11] and used in the EU risk assessment. Although PMF standardised measurements describe the variability of deposition from site to site (variability between geographical locations), they do not reveal the variability of leaf deposition within each site (variability between plants, between leaves, and on leaf surfaces)—an aspect not accounted for in the EU risk assessment sufficiently. As demonstrated by our analysis based on in situ measurements, an assessment considering only site-to-site variability will underestimate both the mean and variability of leaf deposition.

Within-leaf variability of pollen on plant leaves is biologically relevant to the exposure of butterfly larvae to *Bt* pollen. During sensitive early stages, lepidopteran larvae do not generally consume whole leaves, but instead much smaller portions, down to square millimetres; consequently, the distribution and variability of pollen on leaf surfaces is important for estimating the exposure of non-target larvae to *Bt* pollen. As the dose–response relationship of butterfly larvae to *Bt* protein is non-linear [6, 7, 49], within-leaf variability of pollen concentration needs to be acknowledged, with disproportionally higher effects anticipated with increasing pollen densities. Felke and Langenbruch [36] have shown that the consumption of even very small amounts of toxic *Bt* maize pollen may cause lethal effects in sensitive butterfly larvae. These authors reported *Bt* maize pollen (*Bt* 176) LD_50_ values as low as 9 pollen grains/cm^2^ for the fourth larval instar of *Plutella xylostella*. Lethal effects were observed upon a single uptake of four or more *Bt* pollen grains, with earlier instars being even more sensitive. *Bt* varieties differ in the type of *Bt* toxin and show great variation in amount of toxin expressed in pollen. However, only few native butterfly species have been tested to quantify the effects elicited by *Bt* pollen [5].

In this study, individual measurements of pollen leaf density over the flowering period were shown to be log-normally distributed. We have demonstrated that leaf deposition can be related to standardised technical measurements of concentration and deposition, thus allowing a standardised comparison of leaf deposition data. In this regard, the integrated deposition measured by the standardised PMF sampler reflects an average leaf deposition rate over the flowering period. At the same time, it is important to note that leaf deposition is plant-specific and furthermore depends on a wide range of biological and meteorological factors. Consequently, extensive and detailed measurements are required over the flowering period to guarantee a representative assessment of leaf deposition. Because pollen deposition decreases with increasing distance from the source, we measured leaf deposition variability at sites near the source to increase the efficiency of our measurements. Furthermore, leaf deposition data are specific to plant species and site conditions; to obtain comparable results, these data must thus be supplemented by standardised technical measurements to indicate the intensity of airborne pollen deposition at sites.

Our results demonstrate that an integrated monitoring approach is also essential for a correct exposure and risk assessment. Acquisition of exact data on the course and intensity of pollen shedding over the flowering period, accomplished by continuous measurement of within-field pollen concentrations using adequate volumetric pollen monitors that determine pollen release over time, is the base of this approach. Together with meteorological data and field size, these data can represent source strength in suitable dispersal models. After calibration with standardised deposition measurements, a selected model allows pollen deposition over the flowering period to be predicted with high temporal and spatial resolution for standardised average pollen deposition at each location. A more realistic assessment of exposure may be achieved by performing parallel in situ measurements of leaf deposition variability at selected sites for relevant plant species. In our pilot study in Brandenburg, Germany [37, 39], we tried to demonstrate this principle by combining the necessary measurements to follow the release of maize pollen from source fields and its aerial transport and deposition onto leaves at monitored sites.

## Conclusions

Our study is the first reported investigation focused on the variability of maize pollen deposition on plant leaves with a sufficiently high spatial and temporal resolution to assess exposure and thus potential harmful effects on feeding lepidopteran larvae. The high variability of pollen densities observed within leaves has relevant implications for individual lepidopteran larvae feeding on these leaves. Because young and thus especially vulnerable larvae feed only on a small fraction of a leaf, individual exposure is poorly approximated by the mean number of pollen grains deposited on a leaf. Because dose–response relationships for *Bt* toxins are non-linear [6, 7, 49], the variability of pollen deposition within leaves does not just affect individuals; it also influences population estimates of the adverse effects of *Bt* pollen consumed by butterfly larvae.

Our data were taken in situ from maize and five different plant species growing in and alongside maize fields and representing different host-plant species of lepidopteran larvae. Our results clearly demonstrate that the amount and variability of pollen deposition onto plant leaves is considerably higher than previously assumed. This observation applies to both daily mean and (especially) individual values and is of practical relevance for the risk assessment and management of insect-resistant *Bt* maize. Several aspects of our results are particularly valuable.

In the current EU risk assessment, estimates of the exposure of non-target organisms to *Bt* pollen can only be inferred from a limited database and is based on several expert opinions [14]. In our study, we amassed quantitative data consisting of in-field measurements of leaf deposition and its variability and related these data to simultaneous technical pollen sampling as being representative of realistic assessment under common cultivation and for worst-case scenarios. This approach not only allowed an understanding of the distance relationship between pollen deposition and sources; it also provided insight into the nature of the pollen deposition itself as a complex process. The variation in leaf deposition values recorded in our experiments greatly exceeds EU estimates [14], both for realistic and worst-case scenarios. As a consequence, risks due to the exposure of non-target Lepidoptera to *Bt* pollen cannot be excluded. Instead, a revision of the EU model to account for the data presented in this study may be expected to show considerable effects.

Assuming the same mortality model used in the EFSA risk assessment [14] and applying the same protection thresholds, our results regarding exposure under realistic commercial cultivation and worst case imply that buffer isolation distances required to protect sensitive butterfly populations from potential harm should be in the kilometre range instead of the 20–30 m distance defined by the EFSA opinion on risk assessment and management [14].

Simultaneous monitoring of plant-specific leaf deposition along with the measurement of pollen concentration, release rates and pollen deposition by technical sampling can provide deeper insights into processes and relationships. This strategy offers the opportunity to improve models of pollen release and deposition and, more specifically, models of lepidopteran larval exposure to toxic *Bt* pollen and its subsequent effects.

As demonstrated in this investigation, the use of parallel measurements with standardised methods to estimate leaf deposition and exposure can be extended beyond our specific case study to allow a more general assessment under common cultivation conditions.

Methodologically, detailed microscopic in situ measurements of leaf deposition require intense resources and are only efficient close to the pollen source. Their use is necessary in special investigations—for instance, for assessment of leaf deposition variability. For other tasks, including acquisition of comparable data for routine monitoring of pollen deposition, standardised technical measurement methods such as the PMF are more efficient and adequate. In addition, leaf deposition measurements are plant- and site-specific; to generate comparable results, these measurements must therefore be accompanied by standardised technical measurements indicating aerial pollen exposure at a given site.

## Methods

### In situ measurement of pollen deposition on leaves

We applied a new method of in situ measurement of maize pollen deposition described by Hofmann et al. [15] (see Additional file 1: Figure S8). The method allows accurate field identification and quantification of maize pollen on plant leaf surfaces. This approach also enables precise documentation of the process of pollen deposition on plant leaves by repeated measurements of the same plants and leaves over a flowering period. Leaf surfaces were analysed using a digital microscope with a resolution of 1.3 megapixels (Dino-Lite Pro AM413MT; AnMo Electronics Corporation) powered by the USB port of a notebook computer and controlled by DinoCapture 2.0 software. The microscope had built-in LED lights and magnification adjustable from 50 to 200×, thus allowing accurate identification of maize pollen. Each measurement was documented by recording a digital image, and each series was calibrated for exact definition of the evaluation area of each measurement. Quantitative analysis of pollen was conducted visually to ensure correct identification of pollen types and numbers, thereby avoiding errors frequently made by image analysis, such as in cases of pollen overlap or dehydrated pollen.

### Measurement of pollen concentration and deposition by standardised technical sampling methods

For exact assessment of maize pollen release rates, aerial maize pollen concentrations were continuously measured during the flowering period at canopy height in field B using a PMO volumetric pollen monitor (Additional file 1: Figure S9). Details of this method can be found in [10]. The high-volume sampler allowed measurement of pollen concentrations close to the source and even under turbulent wind conditions in the field. Air was sucked into the sampler at a rate of 1000 l/min through an omnidirectional inlet; pollen grains were continuously recorded by impaction on adhesive tape fixed to a rotating drum at a flow rate of 10 l/min, analogous to the principle of the Hirst trap (Sporewatch, Burkard Scientific Ltd.). The height of the inlet was adjusted to match that of the developing plants, thus allowing measurements to be taken continuously at canopy level at the height of the pollen-releasing tassels.

To obtain comparable pollen deposition data at all leaf measurement sites, we additionally used PMF passive samplers (Additional file 1: Figure S10) standardised according to VDI 4330-3 [19] and CEN-TS 18617-1 [47]. Further method details are given in [9].

### Sampling design

To encompass the entire flowering period in the study region, we selected two maize fields with different flowering periods located in the vicinity of Angermünde, Uckermark, Brandenburg, Germany (Additional file 1: Figure S11). Measurements started on 28 July 2010, reflecting the host vegetation, at Hügel (A1) and Soll (A2) subsites of early flowering maize field A (38 ha). On 7 August 2010, following the start of pollen shedding on 3–4 August, measurements began in later-flowering maize field B (30 ha). Daily measurements were taken on maize [A1, A2 and B] and nettle leaves [A1, B], while supplementary measurements of blackberry [A1], sorrel [A2] and goosefoot [B] were obtained on alternate dates. The sampled plant species, which represented different host plants of European butterfly populations, were growing naturally inside fields or at field edges. These plant species were also chosen to reflect different leaf acceptor conditions.

To obtain representative measurements of the variability of pollen deposition on leaf surfaces, we applied a structured sampling design described by [15]. This design ensured that the inhomogeneity and variability of pollen leaf deposition were reflected in both mean and variance. The microscopic device we used allowed for an evaluation area of 0.05–0.5 cm^2^ (i.e. 5–50 mm^2^) per single measurement. Lauber [49] has reported a total leaf consumption area of 0.07 cm^2^ by individuals of European Lepidoptera *Inachis io* in the first larval stage in Hungary. Assuming a 3-day period on average for the first larval stadium, a daily intake of 0.02–0.03 cm^2^ (2–3 mm^2^) can be expected. Our selected evaluation area therefore reflected the feeding behaviour of sensitive first instars of *Inachis io* and was adequately sized to detect the variability of pollen deposition encountered by lepidopteran larvae. We note here that an evaluation area of 1 cm^2^ or even greater, such as whole leaves, found in published studies seems to be too large.

Our structured sampling design consisted of a combination of three transects with five measuring points and four clusters with three measuring points (i.e. a total of 27 points). At each site, three leaves of three plants of each species were examined on each measurement date. This sampling approach proved to be the most effective way to reflect the variability of pollen distribution for in situ measurements.

### Data compilation and statistical analysis

For compilation and statistical analyses of the data, we used Microsoft Excel [50], the statistical tool XLSTAT-Pro 2011 [51] and the open-source software package R [52].

### Leaf deposition

Maize pollen deposition on leaves of individual plant species was quantitatively measured according to the equation

1 $$D_{{{\text{i}},t}} = \frac{{M_{{{\text{i}},t}} }}{{A_{{{\text{i}},t}} }} = D_{{{\text{i}},j(t)}} , \quad {\text{in}}\,{\text{n/cm}}^{2} {\text{(pollen grains per cm}}^{2} ),$$

where *D*_i*,t*_ is a single measurement of plant-specific pollen deposition on the leaf surface at time/date *t* in n/cm^2^ (pollen grains per cm^2^), also known as pollen density and given by the ratio of *M*_i,*t*_ (as the observed maize pollen count) per evaluation area *A*_i,*t*_ on the leaf surface of plant species i at time *t*. Single observations are additionally characterised by the index j, which enumerates all observations over the flowering period.

### Standardisation of leaf deposition data to the same distance from the pollen source

Samples were taken within fields and at field edges. However, deposition declines with increasing distance from the pollen source. For data to be comparable, measurements must be standardised to the same distance. Using the regression function for pollen deposition as measured by the PMF standardised method [9], all measurements were adjusted so that their values corresponded to a within-field location near the pollen source (0.2 m). For instance, measurements taken at a 1-m distance were adjusted by a conversion factor of 2.57.

### Statistical distribution of leaf deposition data

Statistical variation in the density of pollen deposition was analysed in two ways. In addition to describing this variation by the empirical distribution function of the data, we fitted a log-normal distribution to observed pollen densities using the CML estimation method as described below. The major results of this study are derived from this fit. To calculate the empirical distribution, values below the detection limit were replaced by a surrogate value. Such surrogate values, which are somewhat arbitrary, are not required by the CML method. To graphically display pollen deposition distributions, we calculated nonparametric kernel density estimates [53]. For this purpose, values below the detection limit were replaced by random values from a log-normal distribution with parameters estimated by CML. The main parameters used to describe mean location and spread of the data were the geometric mean of the log-normal distribution (identical to its median), which is less susceptible to extreme values than the arithmetic mean, and selected quantiles. The standard deviation is less informative because the distribution of pollen disposition on a linear scale is clearly skewed. The validity of this approach was confirmed by applying this method to the pollen deposition data of maize leaves in field B, where no values below the detection limit had been recorded.

### Empirical distribution

An empirical distribution describes the observed distribution of a raw dataset without assumptions about its mathematical form. Data below the detection limit are replaced by a value corresponding to two-thirds of the detection limit. This surrogate value is based on the assumption that values between the detection limit and zero follow an approximately triangular distribution. The use of a surrogate value for values below the detection limit, frequently done for simplicity, has the disadvantage that the dispersion of the data tends to be underestimated. If the number of values below the detection limit is large, quantiles of interest, such as the 10 % quantile, cannot be calculated in a realistic way.

### CML estimation

The calculation of small quantiles, means and standard deviations is compromised by the presence of values below the detection limit. Selecting surrogate values and treating them like measured values generates biased parameters. This problem can be avoided by using the CML approach for estimating distribution parameters. This approach requires an assumption to be made about the pollen density distribution. Because the data in this study apparently followed a log-normal distribution, we used this distribution for our calculations. A log-normal distribution of the data implies a normal distribution of the logarithmic data. The CML approach uses the pollen density *D*_i*,j*_, where measured, and, if the observed density is only known to lie below this limit (in statistical terms: is censored), the detection limit *ϑ*_i*,j*_. The censoring information is coded by the indicator *c*_i,j_, which has a value of 1 if censoring has occurred and 0 otherwise. The CML approach then chooses those values of µ_log10_ and σ_log10_ as estimates for the mean and standard deviation of the underlying log-normal distribution, which maximises the likelihood function *L. L* is defined by

2 $$L\left( {\mu_{\text{log10}} ,\sigma_{\text{log10}} ;D_{i,j} ,c_{i,j} } \right) = \mathop \prod \limits_{j = 1}^{{J_{i} }} \left[ {p\left( {\log_{10} D_{i,j} ,\mu_{\text{log10}} ,\sigma_{\text{log10}} } \right)} \right]^{{1 - c_{i,j} }} \cdot \left[ {P\left( {\log_{10} \vartheta_{i,j} ,\mu_{\text{log10}} ,\sigma_{\text{log10}} } \right)} \right]^{{c_{i,j} }} ,$$

where i is plant species (acceptor), *j* is the observation, *J*_i_ is the total number of observations for species i, *D*_i*,j*_ is the observed deposition if not censored (otherwise 1), *ϑ*_i*,j*_ is the detection limit for species i and observation j, *c*_i*,j*_ is the censoring indicator (0 if not censored, otherwise 1), µ_log10_ is the mean of the log-normal distribution on a log_10_ scale, σ_log10_ is the standard deviation of the log-normal distribution on a log_10_ scale, *p* is the probability density function of the normal distribution and *P* is the corresponding cumulative density function.

Maximisation of *L* was performed iteratively using the Newton–Raphson algorithm. If no censoring takes place, the solutions of (2) simplify to the well-known standard equations for the empirical mean and standard deviation of log_10_*D*_i*,j*_. The coefficients µ_log10_ and σ_log10_ are on a decadic logarithmic scale. Arithmetic means and standard deviations of the CML estimates on a linear scale are obtained by

3 $$\sigma_{ln} = \ln 10^{{\sigma_{log10} }} ,$$

4 $$\mu_{ln} = \ln 10^{{\mu_{log10} }} ,$$

5 $$\mu_{lin} = \exp \left( {\mu_{ln} + 0.5\sigma_{ln}^{2} } \right),$$

6 $$\sigma_{lin} = \mu_{lin} \sqrt {\exp \sigma_{ln}^{2} - 1} .$$

The median of the log-normal distribution on the log_10_ scale (which coincides with the arithmetic mean, median and mode on that scale) is transferred to a linear scale by

7 $$Median_{lin} = 10^{{\mu_{log10} }} ,$$

where it coincides with the geometric mean.

### Relation of concentration and deposition

The process of particle deposition on an acceptor surface is related to the aerial particle concentration by Stokes’ law via the deposition velocity, *v*_d_, described in (8). Based on the continuous PMO concentration measurements, we calculated the daily deposition rate *D*_d_ using the daily mean concentration and the deposition velocity *v*_D_ according to (9). Deposition velocity for maize pollen varies between 0.15–0.4 m/s [54, 55, 56]. We assumed an average *v*_D_ of 0.2 m/s [9, 57] in our calculations:

8 $$D = C \cdot v_{\text{D}} \cdot \varepsilon \quad {\text{in n}}/{\text{cm}}^{ 2}$$

9 $$D_{\text{d}} = C_{\text{d}} \cdot v_{\text{d}} \cdot \varepsilon \quad {\text{in n}}/\left( {{\text{cm}}^{ 2} \,{\text{day}}} \right)$$

where *D*_d_ is the daily deposition rate on an acceptor surface in n/(cm^2^ day), *C*_d_ is the daily mean concentration in n/m^3^ as measured by the PMO pollen monitor, *v*_D_ is deposition velocity for maize pollen in m/s (0.2 m/s) and ε is a conversion factor (8.64) for the number of seconds in a day (86,400) and for m^2^ to cm^2^ (0.0001).

### Deposition rates and total and standardised deposition

Because deposition is a process, the amount of deposited pollen on a surface at time *t* is the integrated deposition over the time period from the start of pollen shedding until time *t*. This deposition process is expressed by the integral of deposition over this time period and can be calculated on a daily time scale using the respective cumulative daily deposition rates (10). The total or integrated deposition is given by the integral over the whole flowering period or the cumulative deposition of the daily rates, respectively (11).

The integrated potential deposition *D*_pot_ is a special case of *D*_total_ that provides an estimate assuming a permanent downwind position. This parameter has been introduced for considering worst-case assessments depending on wind direction when measurement is carried out adjacent to fields [29, 30].

The deposition measured with a PMF passive sampler standardised according to [19, 47] is the respective standardised integrated deposition *D*_S_ integrated over the exposure period (in our case, flowering period). As deposition is acceptor-specific, this is indicated by the respective index S. The PMF deposition values allow a comparison of the intensity of pollen deposition from site to site [9]. *D*_S_ corresponds to the total deposition based on aerial concentration measurements according to Eq. 12, leading to a conversion factor $\varphi$_S − total_ of 0.191:

10 $$D_{t} = \int\limits_{0}^{t} {D(s)ds} \approx \sum\limits_{s = 1}^{t} {D_{\text{d}} (s)} ,$$

11 $$D_{{{\text{total}}}} = \int\limits_{0}^{T} {D(s)ds} \approx \sum\limits_{{s = 1}}^{T} {D_{{\text{d}}} } (s),$$

12 $$D_{\text{S}} = \varphi_{{\text{S}} - {\text{total}}} \cdot D_{\text{total}} = 0.191 D_{\text{total}} ,$$

where *D*_*t*_ is pollen deposition on the area of the acceptor surface (pollen density) at time *t* in n/cm^2^, *D*_d_ is the daily deposition rate in n/(cm^2^ day), *D*_total_ is the cumulative deposition over the flowering period (n/cm^2^) as measured by the volumetric pollen monitor, *D*_S_ is the standardised deposition over the flowering period (n/cm^2^) as measured by the PMF passive sampling method and $\varphi$_S_ _− total_ is a conversion factor for *D*_S_ versus *D*_total_.

### Relationship of plant-specific leaf deposition to standardised technical measurements

Pollen deposition on leaves is plant-specific and describes the measured pollen grain density on a leaf surface over time. In addition to deposition rates serving as input, translocation processes and losses due to wind and rain influence the measured density on the leaf surface. Thus, leaf deposition is the net result of the processes of pollen deposition and losses on the leaf surface taking place over the whole flowering period. The relationship between specific mean leaf deposition for plant species i and the standardised measurement of total pollen deposition *D*_total_ over the flowering period using the PMO pollen monitor is described by (13)

13 $$\varphi_{{i - {\text{total}}}} = \frac{{\bar{D}_{\text{i}} }}{{D_{\text{total}} }},$$

where $\varphi$_i − total_ is the conversion factor for specific leaf deposition of species i versus total deposition, $\bar{D}_{\text{i}}$ is CML-estimated mean leaf deposition on species i over the flowering period (in n/cm^2^) and *D*_total_ is the total deposition over the flowering period (n/cm^2^) obtained by standardised technical measurement of aerial pollen concentrations using the PMO pollen monitor. The relationship between specific mean leaf deposition for plant species i and standardised deposition *D*_S_ measured by the PMF leads accordingly to the following equation:

14 $$\varphi_{{i - {\text{S}}}} = \frac{{\bar{D}_{\text{i}} }}{{D_{\text{S}} }},$$

where $\varphi$_i − S_ is the conversion factor for specific leaf deposition of species i versus standardised deposition, $\bar{D}_{\text{i}}$ is CML-estimated mean leaf deposition in n/cm^2^ and *D*_S_ is standardised deposition over the flowering period (n/cm^2^) determined by technical sampling using the PMF passive sampler according to VDI 4330-3 [19].

With respect to worst-case assessments, relationships can be evaluated for 90 and 95 % quantiles of leaf deposition values in an analogous way by using the respective quantile values in the above equations instead of the mean leaf value, $\bar{D}_{\text{i}}$.

### Expected variability of leaf deposition data over sites and years inferred by combining site-to-site and within-site variation

The expected variability of leaf deposition data is estimated by combining observed site-to-site variation of standardised measurements of deposition obtained using the PMF as described by Hofmann et al. [9] with observed variation in leaf deposition data at each site as described in this study by the CML. The spread of the 95 % CI indicating the expected variability of leaf deposition data over sites and years around the mean value is calculated for species *i* according to (15):

15 $$CI_{{D{\text{i}}}} (95 \,\% ) = \pm 1.96 \cdot \sqrt {\left( {\sigma_{\text{S}}^{2} + \sigma_{\text{i}}^{2} } \right)} ,$$

where *CI*_*D*i_ (95 %) is the combined 95 % CI for leaf deposition data of species i within and across sites, σ_s_ is the standard deviation of standardised PMF deposition data across sites and σ_i_ is the standard deviation of leaf deposition data for species i within a given site.

## Abbreviations and definitions

A list of abbreviations and definitions is included to clarify terms and parameters used in this paper.

**Table:** 

Accumulation	Increase in the amount of pollen deposited on leaves over time. Accumulation can be described using mean leaf-density values; while the mean value increases over time, the stochastic variability around the mean basically remains stable (see “aggregation”)
Aggregation	Describes the tendency of particles or pollen grains to progressively clump together on leaf surfaces, leading to increased variability in leaf-density values around the mean over time. Aggregation contributes to stochastic variation at the time of deposition during the process of accumulation (see “accumulation”)
*Bt*	*Bacillus thuringiensis*
CA	Index for *Chenopodium album*
CML	Censored maximum likelihood (estimation method)
Cumulative deposition	Sum of daily deposition rates (see “integrated deposition”)
*d*	Index for day/daily
Daily deposition rate, *D*_*d*_	Daily pollen input per surface area from standardised measurements of aerial pollen concentration in n/(cm^2^ day). Obtained with a volumetric pollen monitor.
Daily mean concentration, *C*_*d*_	Daily mean number of pollen grains per air volume in n/m^3^ from standardised measurements using a volumetric pollen monitor.
Daily leaf deposition, *D*_*d,*i_	Mean value of observed pollen density on a leaf surface (n/cm^2^) of plant species i for a given day *d*, averaged over measurements from one day per site
Deposition, *D* *Indices:* i, *j*, *t*, *d*, *T*, total, pot, S, ZM, UD, CA, RA, RU	Describes (a) the process of pollen loading (input) from the air to an acceptor (surface area) as a function of time, *D*_*t*_; (b) the resulting deposition value, *D*_i*,t*_, equivalent to the measured number of particles per surface area of acceptor i (number density in n/cm^2^) at time *t*. Indices are used for standardised technical measurements and plant leaves as acceptors. (See “deposition rate”, “daily deposition rate”, “integrated deposition”, “cumulative deposition”, “total deposition”, “integrated potential deposition” and “standardised deposition”)
Evaluation area, *A*	Leaf area for a single measurement (here, 5–50 mm^2^)
Flowering period, *T*	Period of pollen shedding (here, of maize)
GMO	Genetically modified organism
i	Index for acceptor
Integrated concentration, *C*_total_	Integrated concentration over flowering period *T*, serving as an index of the intensity of pollen flow at a measurement site (= cumulative sum of daily mean concentration values); units of n/m^3^
Integrated deposition, *D*_total_	Total pollen deposition integrated over the entire flowering period *T*, from standardised measurements of air concentration obtained using a PMO pollen monitor; equivalent to cumulative deposition of daily deposition rates over the flowering period and also the final value of deposition *D(t)* over time on a standard acceptor. Units of n/cm^2^
Integrated potential deposition, *D*_pot_	Integrated deposition over the flowering period assuming a permanent downwind position (n/cm^2^)
Integration	(a) Deposition: Integration describes the integral of the results of input–output processes taking place on an acceptor over time in the course of deposition (e.g. pollen density on a leaf surface or pollen captured by a PMF passive sampler). Accumulation, in a strict sense, sums together the input rates, whereas integration refers to the measured density values on the acceptor and also reflects sample efficiency and losses. (b) Concentration: Concentration cannot be summed over time, only averaged. The index of total yearly concentration is calculated as the sum of daily mean concentration values per m^3^ air volume over the flowering period to indicate the intensity of pollen flow at a site; this index might be more correctly described as an integral of concentration over time
*j*	Index for single observation
Leaf deposition, *D*_*i*_	Measured pollen density on the leaf surface of a species *i* at time *t* in n/cm^2^
Mean concentration	Average number of pollen grains in a volume of air per time period, e.g. hourly or 3-hourly (see “daily mean concentration”)
n/m^3^	Pollen grains per cubic metre of air volume
n/cm^2^	Pollen grains per square centimetre
n/(cm^2^ day)	Pollen grains per square centimetre per day
PMF	Pollen mass filter, a passive pollen sampler standardised according to VDI 4330-3 and CEN-TS 16817-1 for integrated measurement of pollen flow and deposition in the air over a measurement period of up to several weeks during flowering
PMO	Pollen monitor, a continuously recording, high-volume aerosol particle sampler with an omnidirectional inlet for standardised measurements of aerial pollen concentration (n/m^3^); (see “mean daily concentration”, “integrated concentration over flowering period”, “deposition”, “daily deposition rates” and “integrated deposition over flowering period”)
Pollen concentration, *C*(*t*)	Number of pollen grains per air volume (n/m^3^) at time *t*, determined in this study by standardised, continuous measurement using a volumetric pollen monitor (see “daily mean concentration”, “integrated concentration” and “deposition”)
Pollen count, *M*	Measured pollen density on a leaf, recorded as the number of pollen grains per evaluation area
pot	Index for potential (see “integrated potential deposition”)
RA	Index for *Rumex acetosa*
Conversion factor, $\varphi$	Factor describing the relation between two pollen exposure parameters, e.g. leaf deposition on *Urtica dioica* with standardised deposition $\varphi_{{{\text{UD}} - {\text{S}}}}$
RU	Index for *Rubus* sp.
S	Index for standardised deposition (see “standardised deposition”)
Standardised deposition, *D*_S_	Standardised measurement of integrated deposition over flowering period *T* obtained by the PMF passive sampling method according to VDI 4330-3 and CEN-TS 16817-1
*t*	Index for time/date
Total	Index for integrated concentration and integrated deposition (see definitions) over the flowering period
UD	Index for *Urtica dioica*
*v*_D_	Deposition velocity in m/s
ZM	Index for *Zea mays*

## Additional file

10.1186/s12302-016-0082-9 Supplementary information (Appendix). **Figures S1–S11** are available as supplementarymaterial to this article.
