# Supplementary material for: Accumulation and variability of maize pollen deposition on leaves of European Lepidoptera host plants and relation to release rates and deposition determined by standardised technical sampling
Source: Environ Sci Eur. 2016 Apr 26;28(1):14. doi: 10.1186/s12302-016-0082-9 (PMC5044972; doi:10.1186/s12302-016-0082-9)
Supplement: Supplementary file 1 — 10.1186/s12302-016-0082-9 Supplementary information (Appendix). Figures S1–S11 are available as supplementarymaterial to this article. [file 12302_2016_82_MOESM1_ESM.docx]

# Additional file 1: Supplementary information

Figure S1 (Inhomogeneous distribution of pollen…)

Figure S2 (Examples of deposited pollen on maize leaves…)

Figure S3 (Maize pollen deposition on Urtica dioica leaves…)

Figure S4 (Maize pollen deposition on leaves of Chenopodium album…)

Figure S5 (Probability density distribution of maize pollen deposition…)

Figure S6 (Cumulative distribution of maize pollen …)

Figure S7 (Comparison of leaf deposition with daily mean deposition rates…)

Figure S8 (In situ digital microscopy…)

Figure S9 (PMO pollen monitor for continuous measurement…)

Figure S10 (PMF passive pollen sampler…)

Figure S11 (Measurement sites…)

**Figure S1 Inhomogeneous distribution of pollen on maize leaves.** Areas of accumulation and aggregation are illustrated in photographs as follows: (a) after the start of maize flowering, 28 August 2010 at site A1; (b) after the start of maize flowering, 28 August 2010 at site A1; (c) upper leaf during main pollen shedding, 6 August 2010 at site B; (d) accumulation at the leaf base after rain, 7 August 2010 at site B; (e) accumulation and aggregation on the leaf surface during main pollen shedding, 10 August 2010 at site B; (f): aggregation of pollen away from the midrib, 10 August 2010 at site B.

**
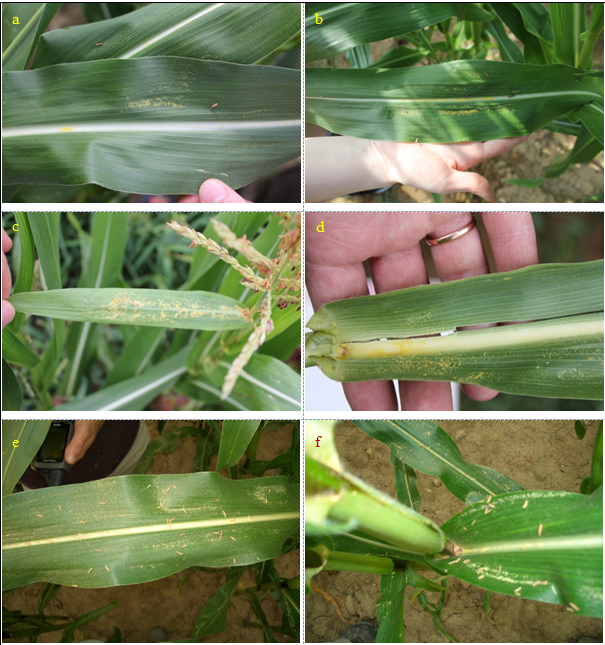
**

**Figure S2** **Examples of deposited maize pollen on maize leaves.** (a) Leaf, 200×, 28 July 2010, site A1, density = 162 grains/cm²; (b) leaf, 200×, 28 July 2010, site A1, density = 1,368 grains/cm²; (c) leaf with marked pollen and lines to assist quantification, 200×, 28 July 2010, site A1, density = 6,822 grains/cm²; (d) leaf base, 50×, 12 August 2010, site B. An exact density measurement was not possible in the case of (d); instead, an estimate was made via a pollen count on the surface layer multiplied by an estimate of the number of pollen layers that could be observed in the crack: the aggregation area in the image was >1 cm², implying a pollen density of >103,000 grains/cm².


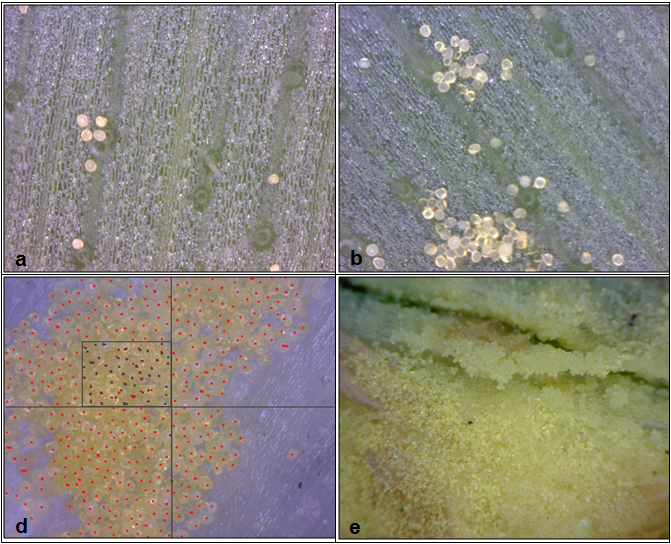


**Figure S3** **Maize pollen deposition on *Urtica dioica* leaves during the 2010 maize flowering season.** Shedding in field A from the earlier-flowering variety started on 23 July and in field B from the later variety on 3–4 August. The maize flowering period ended around 23 August. Leaf deposition measurements were made in 2010 in field A from 28 July to 6 August and in field B from 7 to 17 August. Values were standardized to a reference point close to the source (0.2 m). Green dots, data (*n* = 1,646); solid red line, daily mean; red dashed lines, 90 % and 10 % quantiles based on the daily data and the log-normal distribution fitted by censored maximum likelihood.


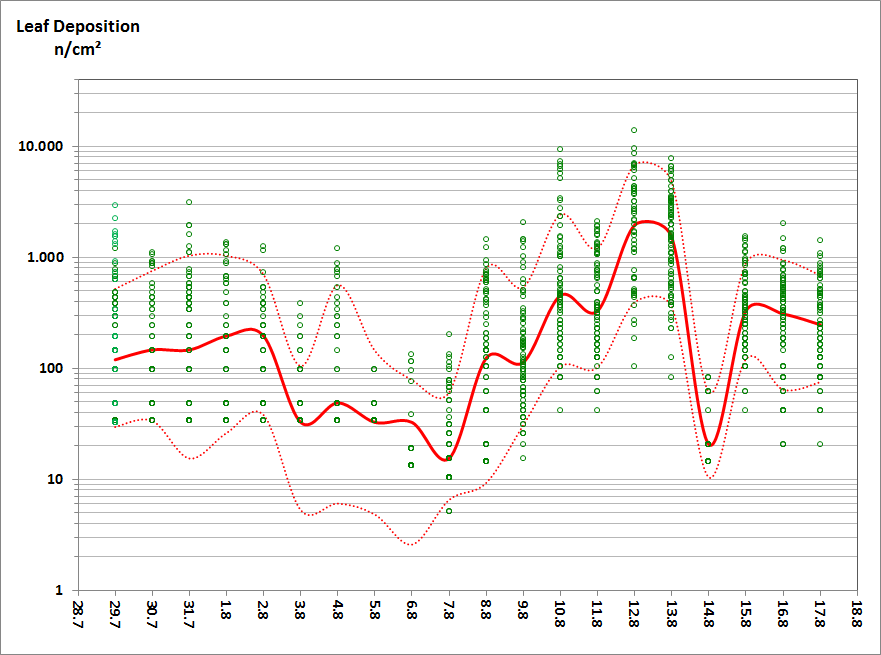


**Figure S4** **Maize pollen deposition on leaves of *Chenopodium album*, *Rumex acetosa* and *Rubus* sp. during the 2010 maize flowering season.** Shedding in field A from the earlier-flowering variety started on 23 July and in field B from the later variety on 3–4 August. The maize flowering period ended around 23 August. Leaf deposition measurements were made in 2010 in field A from 28 July to 6 August and in field B from 7 to 17 August. Green, *Chenopodium album* (*n* = 586); blue, *Rumex acetosa* (*n* = 324); red, *Rubus* sp*.* (*n* = 324). Values were standardised to a reference close to the source (0.2 m). Solid red line, daily mean; red dashed lines, 90 % and 10 % quantiles for each plant species based on the daily data and a log-normal distribution.


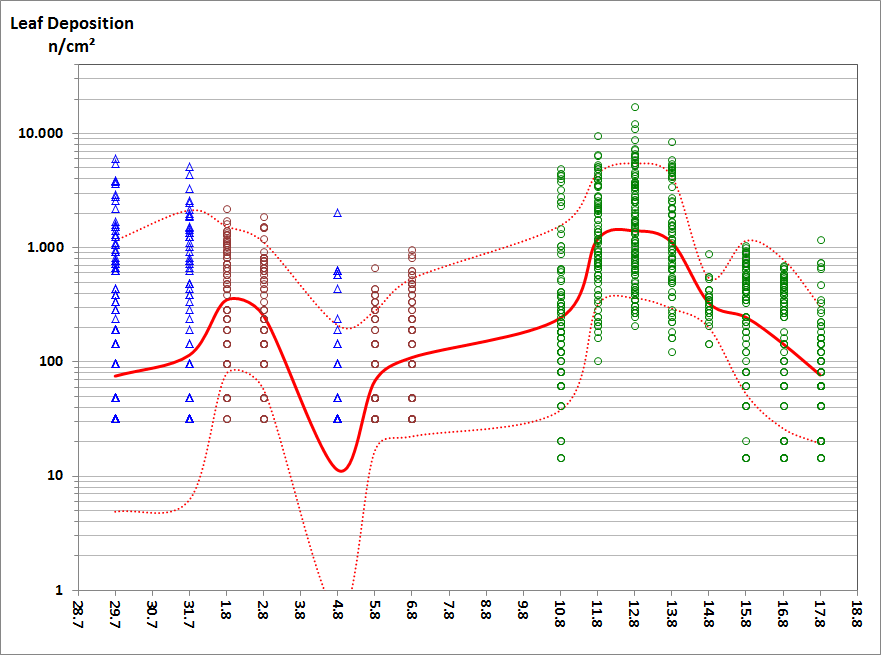


**Figure S5** **Probability density distribution of maize pollen deposition onto plant leaves**. (a–b) Deposition onto maize leaves based on complete data from fields A and B (a) and from field B (b); (c–d) Deposition onto nettle leaves based on complete data from fields A and B (c) and from field B (d); (e–g) Deposition onto leaves of goosefoot (e), blackberry (f) and sorrel (g). Horizontal axis: pollen deposition in n/cm² (log_10_ scale); vertical axis: probability density of pollen deposition, scaled for use with decadic logarithms of deposition. Red solid line, log-normal probability density (censored maximum likelihood estimate); dashed red lines, 10 % quantile, mean (= median) and 90 % quantile; blue solid line, nonparametric probability density (kernel density estimate).

**
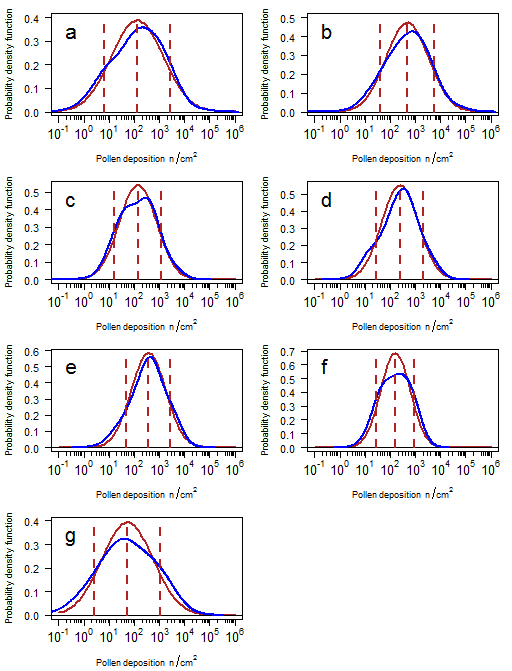
**

**Figure S6** **Cumulative distribution of maize pollen deposition onto plant leaves.** (a–b) Deposition onto maize leaves based on complete data from fields A and B (a) and field B (b); (c–d) Deposition onto nettle leaves based on complete data from fields A and B (c) and from field B (d); (e–g) Deposition onto leaves of goosefoot (e), blackberry (f) and sorrel (g). Horizontal axis: pollen deposition in n/cm² (log_10_ scale); vertical axis: cumulative probability density function of pollen deposition, probability density estimated on log_10_-scale. Red solid line, log-normal distribution (censored maximum likelihood estimate); dashed red lines, 10 % quantile, mean (= median) and 90 % quantile; blue solid line, nonparametric probability distribution function (kernel density estimate).


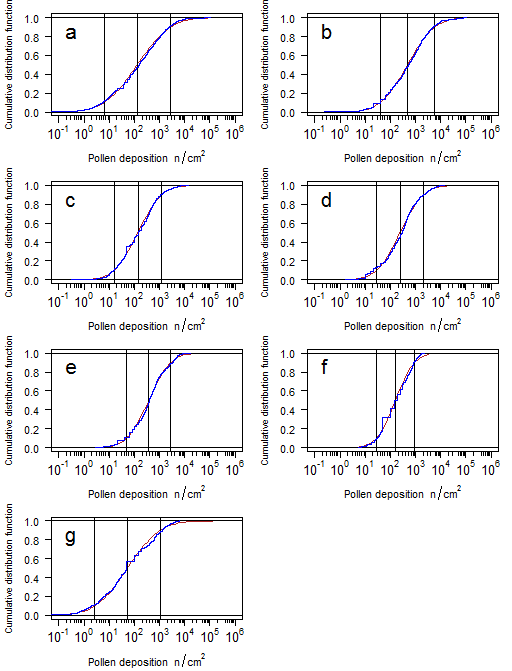


**Figure S7** **Comparison of leaf deposition with daily mean deposition rates, cumulative deposition and PMF-measured integrated deposition over the flowering period.** Data are from late-flowering field B. Parallel measurements of leaf deposition onto maize leaves were made of pollen concentration with a PMO volumetric pollen monitor for release and deposition rates and of integrated deposition with a standardised PMF passive pollen sampler. Dashed line, daily mean deposition rates (maximum 678 n/cm²); blue line, cumulative deposition (total 3,404 n/cm²); red line, daily mean leaf deposition (maximum 2,810 n/cm², overall mean 478 n/cm²); cyan box, PMF-based integrated deposition (359 n/cm²).


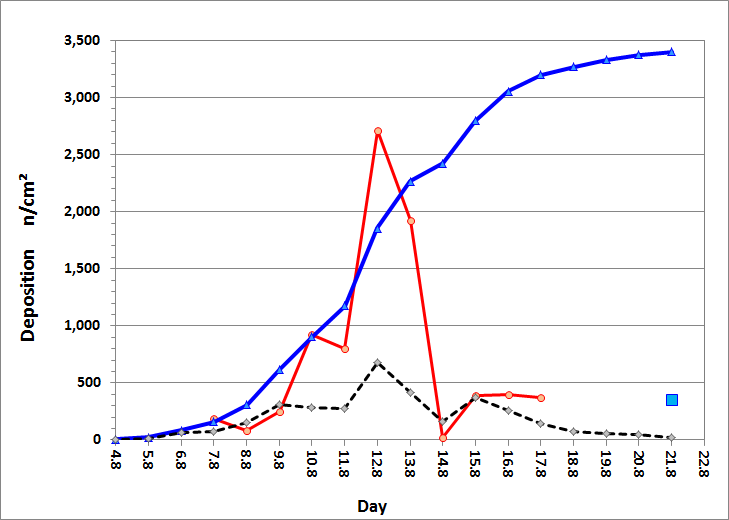


**Figure S8** ***In situ* digital microscopy.** Left-hand side: Dino-Lite Pro system in the field. Right-hand side: *In situ* image (200×) of the upper side of a *Centaurea scabiosa* leaf taken through the Dino-Lite microscope. In this image, maize pollen can be distinguished from other pollen species by its relatively large size (80–120 μm), colour, shape and exine structure. Measurements recorded on the image were calculated by DinoCapture software. (This figure taken from Figs. 1–2 of Hofmann et al. [10]).


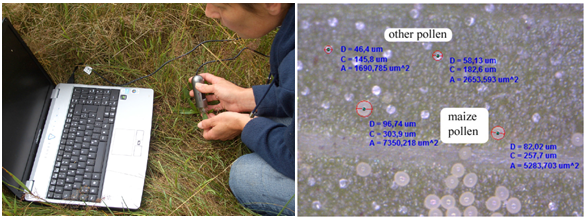


**Figure S9** **PMO** **pollen monitor for continuous measurement of pollen concentration to assess pollen release rates.**  The PMO is a continuously recording, volumetric pollen monitor with an omnidirectional inlet and is suitable for canopy-height measurements inside fields.


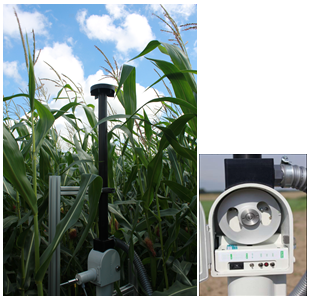


**Figure S10** **PMF passive pollen sampler for standardised measurement of pollen deposition.**

**
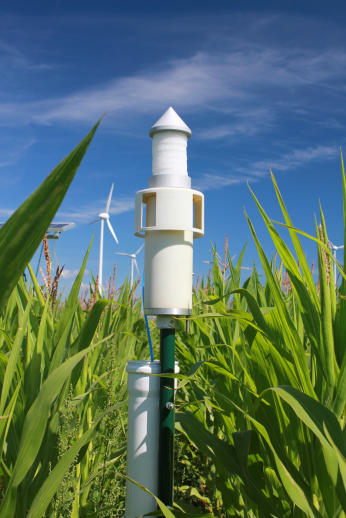
**

**Figure S11** **Sites of measurements of maize pollen leaf deposition.** Earlier-flowering field A with measurement subsites A1 and A2 and the later-flowering field B sampling site are highlighted in orange. Other nearby maize fields are highlighted yellow.

**
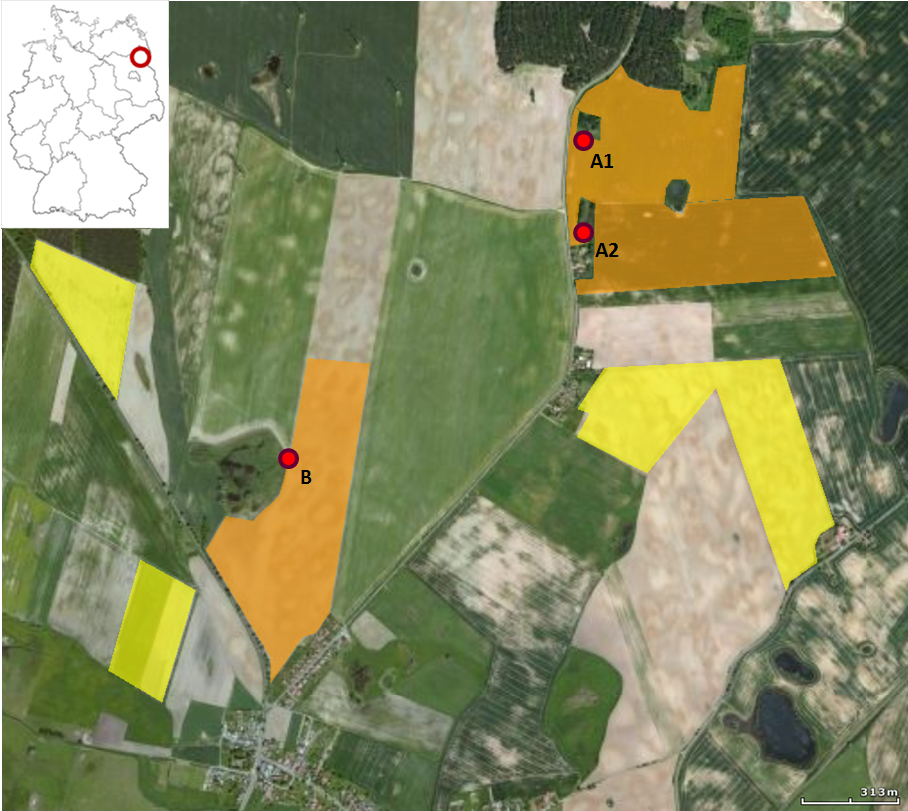
**
